# Differences of Pine Wood Nematode (*Bursaphelenchus xylophilus*) Developmental Stages under High-Osmotic-Pressure Stress

**DOI:** 10.3390/biology13020123

**Published:** 2024-02-16

**Authors:** Shuting Wang, Qiaoli Chen, Feng Wang

**Affiliations:** 1 Key Laboratory of Alien Forest Pests Monitoring and Control-Heilongjiang Province, School of Forestry, Northeast Forestry University, Harbin 150040, China; 1639788062@nefu.edu.cn; 2 Key Laboratory of Sustainable Forest Ecosystem Management-Ministry of Education, Northeast Forestry University, Harbin 150040, China; 3 State Key Laboratory of Tree Genetics and Breeding, Northeast Forestry University, Harbin 150040, China

**Keywords:** *Bursaphelenchus xylophilus*, pine wood nematode, osmobiosis, hypertonic osmotic stress

## Abstract

**Simple Summary:**

Pine wilt disease is a devastating disease, and its causal pathogen, pine wood nematode (PWN), is extremely challenging to manage. This study identified that PWN enters cryptobiosis under high-osmotic-pressure stress. The phenotypic changes and survival of the nematodes during each developmental stage were significantly different. Among them, the third-stage dispersal juvenile had the highest survival and body length change rates. By comparing the gene expression differences between the third-stage dispersal juvenile and other stages, it was found that they exhibited expression differences in genes in energy compound synthesis and anti-reversal signal pathway. The results of this study provide a theoretical basis for the prevention and treatment of PWN by exploiting the differences in high osmotic pressure tolerance at different developmental stages.

**Abstract:**

Under ion imbalance, water deficiency, and salt stress, the osmotic pressure of the tree sap increases, and pine wood nematodes (*Bursaphelenchus xylophilus*, PWN) parasitizing in the trees may be subjected to high-osmotic-pressure stress. KCl, L-malic acid, sucrose, and glycerol solutions were used as osmolytes to explore the highest osmotic concentration that PWN can tolerate. Survival analysis showed that when the treatment concentration exceeded 90%, only a few nematodes in the glycerol group survived under 6 h treatment, and most of the survivors were third-stage dispersal juveniles (DJ3). Further examination revealed that under different concentrations of glycerol-induced high osmotic pressure, the survival rate and body length change rate were the highest in the DJ3 and the lowest in the second-stage propagative juveniles. In order to explore the molecular mechanism of resistance of DJ3 to high osmotic stress, transcriptome sequencing was performed at each developmental stage of PWN and differentially expressed genes that were up-regulated or down-regulated only in DJ3 were screened. The expression of genes related to CoA in DJ3, a key enzyme in metabolism, was significantly higher than the other developmental stages. In addition, the expression of the anti-reversal signal pathway-related gene *AKT-1* in DJ3 was significantly lower than in the other development stages. Therefore, the specific expression of genes in DJ3 under high osmotic pressure may help them rapidly produce and accumulate energy-related compounds and activate the adenosine 5′-monophosphate (AMP)-activated protein kinase (AMPK) pathway to respond to damage caused by high-osmotic-pressure stress in time, thus promoting survival.

## 1. Introduction

Pine wilt disease is a devastating disease that causes severe damage to the ecological environment, forest resources, and forestry economy [1]. In 1972, Mamiya and Kiyohara identified pine wood nematode (PWN, *Bursaphelenchus xylophilus* (Steiner & Buhrer 1934) Nickle 1981) as the pathogen responsible for pine wilt disease and confirmed its pathogenicity [2]. PWN mainly harms *Pinus* species and a few non-pine coniferous trees [3]. After infecting trees, PWN infection symptoms differ based on the tree species, tree age, and environmental conditions. Symptoms include dying in the same year as or in the following years after infection, which indicates that PWN does not always kill trees immediately after they infect them. PWN need a certain period to survive and reproduce before causing tree death [4]. Trees are also exposed to different adverse conditions, such as drought and low temperatures, while they are infected by the PWN. The primary mechanism for trees to cope with adverse stresses is osmoregulation [5].

There are differences in the osmotic adjustment methods of trees under different adversity stress. Trees possess two main mechanisms for regulating osmoregulation under drought stress. The first is through reducing intracellular water content and cell volume, increasing intracellular solutes to maintain osmotic equilibrium, and enhancing water retention capacity [6]. The second is by increasing the osmotic adjustment compounds so that they, as a solvent instead of water, participate in biochemical reactions [7]. However, under low-temperature stress, trees mainly reduce the cellular water by osmoregulation and increase osmotic adjustment compounds which makes the liquid environment in the trees form a glass phase that prevents the cells from freezing [8] to avoid damage to the body [9]. In conclusion, plants usually use osmoregulation to regulate their water potential and increase osmotic adjustment to improve their osmotic pressure to resist adversity stress [10]. This change in the water physiology of trees under stress conditions also has implications for PWN parasitized within the tree. PWN feeds from the xylem parenchyma of host plants. Therefore, PWN’s development needs to adapt to the high osmotic pressure environment caused by water potential variation in plants to ensure survival and reproduction. However, there is a lack of clarity on how PWN tolerates environments with high osmotic pressure and the maximum osmotic pressure they can tolerate.

At present, it has been observed that many nematodes can survive hypertonic osmotic stress by entering a state of osmobiosis (cryptobiosis) [11]. Under osmobiosis, the metabolic activity of nematodes under high osmotic pressure is greatly reduced, and even life is suspended [12], and the metabolic activity of the nematodes is regained when the osmotic pressure is restored to a suitable level for survival [13]. Previous studies found that *Aphelenchoides besseyi* (Christie, 1942) survived under high osmotic pressure by entering osmobiosis [14]. Entomopathogenic nematodes can use osmobiosis to increase their survival rate during the preservation process [15]. *Steinernema carpocapsae* (Weiser, 1995) maintains a high survival rate under high concentrations of the nonionic solvents glycerol and polyethylene glycol through osmotic adjustment [16]. In addition, some soil nematodes also have high tolerance in environments with high osmotic pressure [17]. However, there are few studies on the osmobiosis adaptation mechanism in PWN [18], and the maximum osmotic concentration that PWN can tolerate is still unknown.

Moisture in trees varies from season to season, and their osmotic pressure varies with environmental conditions and in different parts of the body, making it difficult to measure accurately. Osmotic adjustment compounds in plants are mainly divided into two categories: organic solutes synthesized intracellularly (organic acids, soluble sugars, soluble proteins, etc.), and inorganic ions (K^+^, Na^−^, Cl^−^, Ga^2+^, etc.) [19]. In addition, glycerol is an effective protective agent often used to preserve cells and nematodes [20]. Some entomopathogenic nematodes exhibited high survival rates in high concentrations of glycerol [15]. Therefore, in this study, osmotic adjustment compounds (KCL, L-malic acid, sucrose) and glycerol were selected as the osmolytes, and gradient solubilities were set up to investigate the phenomenon of osmobiosis, and the maximum osmotic pressure concentration that can be tolerated by PWN; these were also set up in order to analyze the differences in the ability of PWN to enter into osmobiosis and to tolerate osmotic pressure across different developmental stages. The study’s results will provide a theoretical basis and research foundation for the control of PWN by interfering with the osmotic pressure tolerance of PWN.

## 2. Materials and Methods

### 2.1. Nematode Culture

*Botrytis cinerea* Pers. (1794), which was used to cultivate nematodes, was provided by Key Laboratory of Alien Forest Pests Detection and Control-Heilongjiang Province (College of Forestry, Northeast Forestry University, Heilongjiang, China). The PWN strains used in the experiment were collected from Fushun city, Liaoning Province, China, on 10 August 2017. The PWN was cultured on the lawn of *B. cinerea* at different temperatures in the dark for different periods to obtain results from different stages [21].

### 2.2. Phenotypic Changes of PWN under Different Osmolytes

Mixed-age nematodes were obtained by isolating PWN using the Baermann funnel method. Among them, PWNs in the second-stage propagative juvenile state (J2) accounted for 8%, PWNs in the third-stage propagative juvenile state (J3) accounted for 25%, PWNs in the fourth-stage propagative juvenile (J4) accounted for 24%, and PWNs in the third-stage dispersal juveniles state (DJ3) accounted for 15% of the nematodes. Males accounted for 12%, and in the females accounted for 16% of the total, respectively (the proportion of nematodes in each stage is the same below). Saturated solutions of KCl (Sinopharm Chemical Reagent, Shanghai, China, 20170724-500 g) [18], L-malic acid (Shanghai Macklin Biochemical, Shanghai, China, L813179-100 g) [22] and sucrose (XiLong Scientific, Guangdong, China, 15700101-500 g) [18] were prepared at 25 °C. Saturated KCl, L-malic acid, sucrose, and glycerol solutions (Yongda reagent, Tianjin, China, 20230216-500 mL) [20] were used as osmolytes and gradient concentrations were set at 10%, 20%, 30%, 40%, 50%, 60%, 70%, 80%, 90%, and 100%, respectively. The mixed-age nematode population (300 nematodes in total) was subjected to high osmotic pressure at 25 °C for 6 h. A corresponding population treated with distilled water was used as a control for comparison, and the experiment was repeated three times. The nematodes treated with different concentrations of each osmolyte, and the nematodes treated with distilled water were divided into two groups. In group one, a SZ61 stereomicroscope (Olympus, Tokyo, Japan) and a DM500 microscope (Leica microscope, Wetzlar, Germany) were used to observe and photograph the treated nematodes. In group two, the dehydration-treated nematodes were washed with distilled water to remove the osmolytes from the body surface and rehydrated in distilled water for 12 h, and then observed and photographed.

### 2.3. Survival Rate of PWNs under Different Osmolytes Treatments

Saturated KCl, L-malic acid, sucrose, and glycerol solutions were used as osmolytes and gradient concentrations were set at 10%, 20%, 30%, 40%, 50%, 60%, 70%, 80%, 90%, and 100%, respectively. The mixed-age nematodes (300) were subjected to high osmotic pressure at 25 °C for 6 h. The nematodes treated with different concentrations of each osmolyte and those treated with distilled water were divided into two groups for methyl blue (Shanghai Macklin Biochemical, Shanghai, China, M812703-25 g) staining experiments [23] and rehydration experiments. In group one, the treated nematodes were washed with distilled water, stained with 0.5% methyl blue for 20 min, and then observed and counted with a DM500 microscope. Methyl blue can stain dead nematodes while living nematodes cannot be stained. The number of non-stained nematodes was counted using the DM500 microscope, and the nematode survival rate after 6 h of hypertonic dehydration treatment was calculated. In group two, the dehydration-treated nematodes were washed with distilled water to remove the osmolytes from the body surface and rehydrated in distilled water for 12 h. After 12 h, the number of living nematodes was counted, and the survival rates were calculated.

### 2.4. Phenotypic Changes of PWNs at Different Developmental Stages

Changes in nematode phenotypes and survival rates were observed after treatment with each osmolyte, and glycerol was identified as a more suitable osmolytes for studying the phenomenon of diazotrophic cryptobiosis of PWN. Additionally, glycerin was found to be harmless to nematodes [24]. Therefore, glycerol was utilized to study the adaptation of various developmental stages of PWN under high osmotic pressure. The optimal osmolytes concentrations were set at 10%, 20%, 30%, 40%, 50%, 60%, 70%, 80%, 90%, and 100%. Mixed-age PWNs (300 nematodes in total) were subjected to high osmotic stress treatment at 25 °C for 6 h and 12 h. Three sets of biological replicates were set up with distilled water as control. The nematodes were observed using a SZ61 dissecting microscope. Photographs were taken at 5x magnification to measure the body length and width of the PWNs at each developmental stage after 6 h and 12 h of high osmotic pressure treatment.

### 2.5. Survival Rate Changes of PWNs at Different Developmental Stages

The optimal osmolytes concentrations were set at 10%, 20%, 30%, 40%, 50%, 60%, 70%, 80%, 90%, and 100%. Mixed-age PWNs (300 nematodes in total) were subjected to high osmotic stress treatment at 25 °C for 6 h and 12 h. PWN after 6 h and 12 h of high osmotic pressure treatment were washed with distilled water to remove osmolytes from the body surface and then placed in distilled water for 12 h of rehydration. Survival rates of each developmental stage of PWNs were counted after 6 h and 12 h of high osmotic pressure treatment after subsequent rehydration.

### 2.6. Total RNA Isolation and Digital Gene Expression (DGE) by Sequencing

The Baermann funnel method was used to obtain mixed-age nematodes, which were washed with diethyl pyrocarbonate and centrifuged to remove the clear supernatant. Then, nematodes were frozen (−80 °C) in a mortar with liquid nitrogen and ground to a powder using a pestle. Total RNA was extracted from the powder using TRIzol (Invitrogen, Commonwealth of Massachusetts, Boston, MA, USA, cat. no. 15596-026) [25]. Amplification grade DNase I (Invitrogen, Commonwealth of Massachusetts, USA, catalogue number: 18068-015) was used to remove genomic DNA. The RNA Nano 6000 Assay Kit of the Agilent 2100 Bioanalyzer system (Agilent, Santa Clara, CA, USA) was used to examine the concentration, the RNA integrity number (RIN), and the 28 S/18 S fragments of the total RNA. The purity of the RNA was assessed by NanoDrop™ (Thermo Scientific, Waltham, MA, USA). The high-quality RNA samples were used to construct a cDNA library, and expression profiling was performed by sequencing by the BGISEQ-500 RNA-seq platform (BGI, Shenzhen, China) [26], with 50 bp single-end (SE) reads generated. SOAPnuke (v1.5.2, https://github.com/BGI-flexlab/SOAPnuke, 31 August 2023) was used to filter raw reads to obtain clean reads. Bowite (v2.2.5, http://bowtie-bio.sourceforge.net/Bowtie/index.shtml, 13 October 2023) was used to align clean reads to reference sequences [27]. Clean reads were aligned to the reference genome of PWN (BioSample: SAMEA2272519, http://www.ncbi.nlm.nih.gov/assembly/310678, 14 November 2013) by HISAT (v2.0.4, http://ccb.jhu.edu/software/hisat2/manual.shtml, 7 September 2015) [28]. RSEM (v1.2.12, http://deweylab.biostat.wisc.edu/RSEM, 14 February 2020) was used to calculate and normalize the matched reads to the fragments per kilobase of the exon model per million mapped fragments (FPKM) [29].

### 2.7. Differentially Expressed Genes (DEGs) Screening and Gene Enrichment Analysis

Based on the gene expression differences in the OmicShare tools (www.omicshare.com/tools, 25 October 2021), DEGs were identified by edgeR [30], controlling for the false discovery rate (FDR). The default parameters of edgeR were used. Up-regulated DEGs and down-regulated DEGs only in DJ3 were selected according to log2(DJ3/other developmental stages) ≥ 1 or <−1 (FDR < 0.05). The Kyoto Encyclopedia of Genes and Genomes (KEGG) is the major public pathway-related database [31]. Through KEGG enrichment of up-regulated or down-regulated DEGs in DJ3, the major enriched biochemical metabolic pathways and signal transduction pathways were identified. The analysis results were extracted, and a *Q*-value ≤ 0.05 after a correction by FDR was used as the threshold. Hypergeometric distribution was used to calculate the terms and pathways enriched among the DEGs. The top 20 terms were extracted if more than 20 terms were identified.

### 2.8. RT-qPCR

The Stratagene Mx3000P qPCR system (Agilent, Santa Clara, CA, USA) and the GoTaq 2-Step RT-qPCR System Kit (Promega, Madison, WI, USA, cat. no. A6010) were used for RT-qPCR to determine the expression of candidate genes, with the 18 ribosomal RNA used as the internal control. The PCR program was 95 °C for 10 s and 40 cycles of 95 °C for 5 s, 58 °C for 30 s, and 72 °C for 30 s. The primers used in this study are listed in Table S1. Three independent biological replicates were performed for each treatment, with three technical replicates for each biological replicate.

### 2.9. Statistical Analyses

The standard deviations and significance of differences for survival rates and body length changes were calculated using SPSS software (version: 26.0; https://www.ibm.com/spss, 17 May 2019, Chicago, IL, USA). The differences (*p* < 0.05) between treatment groups of each concentration were indicated by different alphabetical letters, and the differences (*p* < 0.05) between the survival rates of dehydrated and rehydrated nematodes in the same concentration treatment group are indicated by “*”. The RT-qPCR results were normalized as log_2_ (DJ3/JX). The normalization of the data was performed following the GoTaq 2-Step RT-qPCR System Kit and the 2^−∆∆CT^ method [32]. Significance was determined by Student’s *t*-test.

## 3. Results

### 3.1. Osmobiosis of PWN under Different Osmolytes Treatments

Different osmolytes reduced the activity of PWN at different concentrations after 6 h of high osmotic pressure treatment. With the increase in the concentration of osmolytes, nematode activity gradually decreased. After a certain concentration, the nematodes eventually stopped moving. The nematodes treated with L-malic acid stopped their activity at a low concentration of 20%. The nematodes treated with sucrose stopped their activity when the concentration reached more than 40%. However, in the KCl and glycerol treatments, nematodes did not stop their activity until the concentration reached more than 60%.

**Figure 1:**
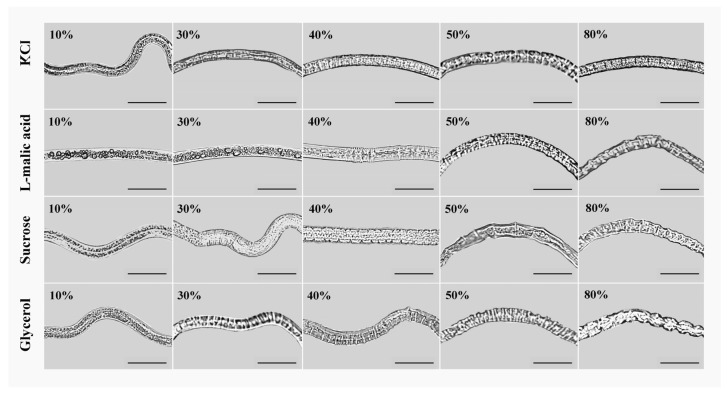
Phenotypic changes of male PWNs after high osmotic pressure treatment with different concentrations (10%, 30%, 40%, 50%, and 80%) of four types of osmolytes for 6 h. Scale bars: 100 μm.

The concentrations at which PWNs showed wrinkling varied among different osmolytes. The phenotypic changes in PWNs treated with different osmolytes exhibited different trends. When the concentration was 10–20%, the nematodes did not shrink after treatment with the four types of osmolytes. When the concentrations were 30%, except for the L-malic acid treatment group, the nematodes in each treatment group began to shrink. Among them, the shrinkage of the nematodes in the glycerol treatment group was obvious, while it was only slightly shrunk in the KCl and sucrose treatment groups. When the concentration reached 40–50%, shrinkage of nematodes in each treatment group was highly apparent. Among them, the nematodes began to shrink at an L-malic acid concentration of 40%. When each osmolyte concentration reached 60–80%, the nematodes in each treatment group continued to shrink, but this trend decreased. After the treatment concentration exceeded 80%, the shrinkage of nematodes in each treatment group no longer changed significantly (Figure 1, the males were shown in the illustration as they had the most obvious phenotypic changes).

By observing the shrinkage of PWN, we found that the KCl treatment group resulted in the smallest changes, while the glycerol treatment group resulted in the most significant changes (Figure 1). In addition, many large-volume lipid droplets appeared in the nematodes treated with L-malic acid at a concentration of 10%. In comparison, large-volume lipid droplets could only be observed in the nematode body when the concentration of the other three osmolytes exceeded 50%. The size of lipid droplets in the body was small, and their number was low (Figure 1). The body fat content of PWNs was different at different developmental stages. The body fat content of males, females, and DJ3 was high, and the change regarding the appearance of lipid droplets was obvious.

**Figure 2:**
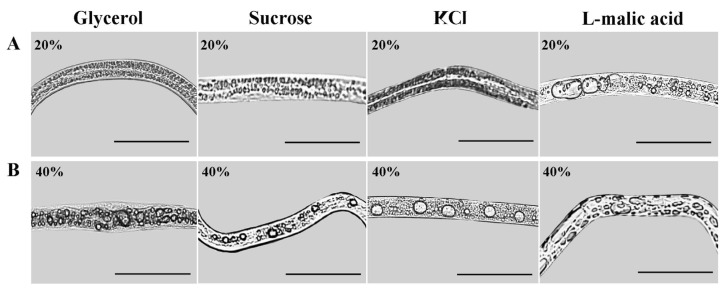
Phenotype of male PWNs after 6 h of high osmotic pressure treatment and 12 h of rehydration. (**A**) Changes in lipid droplet occurrence and properties in males after 6 h of high osmotic pressure treatment with four types of osmolytes (treatment concentration of 20% for each osmolyte) and rehydration for 12 h. (**B**) Changes in lipid droplet occurrence and properties in males after 6 h of high osmotic pressure treatment with four types of osmolytes (treatment concentration of 40% for each osmolyte) and rehydration for 12 h. Scale bars: 100 μm.

After 12 h of rehydration of nematodes treated with high osmotic pressure for 6 h, the body surface of the rehydrated nematodes was stretched, the shrunken body returned to normal, the movement speed was restored, and the partially inactive nematodes were revived. Compared with the high osmotic stress conditions, the number and volume of large-volume lipid droplets in nematodes increased after rehydration. However, the occurrence and properties of lipid droplets in nematodes after rehydration were related to the type and concentration of osmolyte. Large-volume lipid droplets were observed in nematodes in all L-malic acid treatment groups. In contrast, large-volume lipid droplets were observed in nematodes in the other three osmolyte treatment groups only when the treatment concentration reached 20% (Figure 2A, the males were shown in the illustration as they had the most obvious phenotypic changes). The volume of lipid droplets increased with the increase in the osmolyte concentration. When the osmolyte concentration reached 40%, the volume of lipid droplets in nematodes reached the maximum amount and did not change further (Figure 2B). However, the changes in lipid droplets in nematodes in the L-malic acid treatment group were more significant compared to the other osmolyte treatment groups after rehydration (Figure 2).

### 3.2. Survival Rate of PWNs under High Osmotic Pressure Treatment with Different Osmolytes

**Figure 3:**
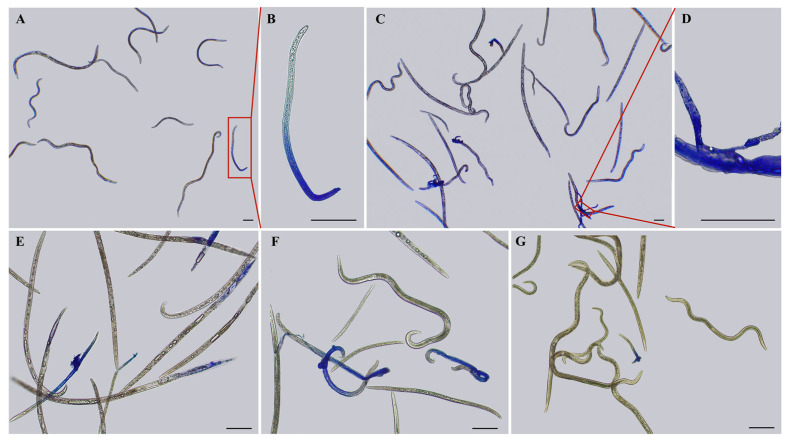
Staining and survival rate of PWNs under high osmotic pressure treatment with different osmolytes for 6 h. (**A**) Staining of nematodes treated with a 20% saturated KCl solution for 6 h. The red line and the red box are circled for the enlarged view, i.e., Figure B. (**B**) Staining of nematodes treated with a 20% saturated KCl solution for 6 h. (**C**) Staining of nematodes treated with a 40% saturated KCl solution for 6 h. The red line and the red box are circled for the enlarged view, i.e., Figure D. (**D**) Staining of nematodes treated with a 40% saturated KCl solution for 6 h. (**E**) Staining of nematodes treated with a 20% saturated L-malic acid solution for 6 h. (**F**) Staining of nematodes treated with a 20% saturated sucrose solution for 6 h. (**G**) Staining of nematodes treated with a 20% saturated glycerol solution for 6 h. Scale bars: 100 μm.

**Figure 4:**
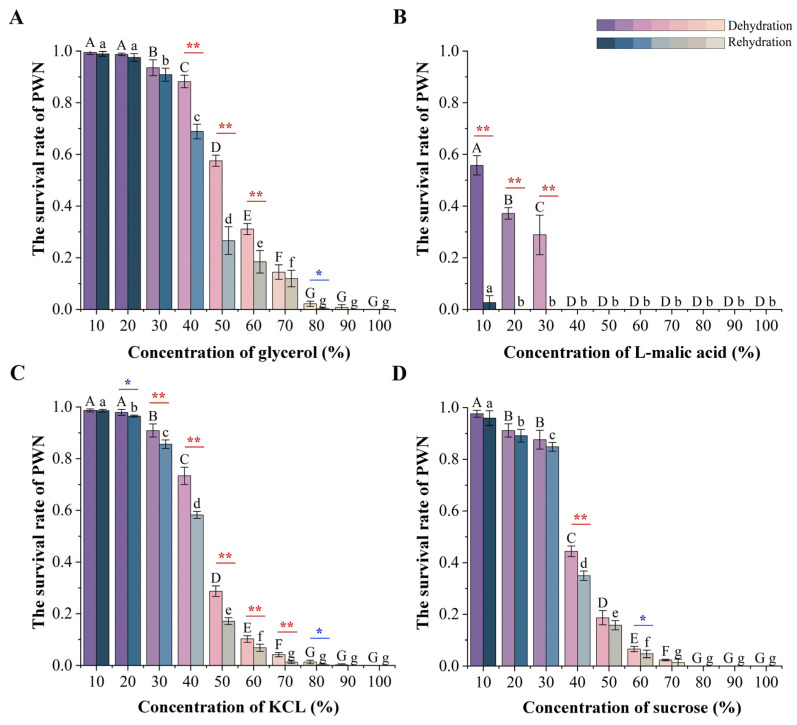
The survival rate of PWNs treated with four types of osmolytes after 6 h dehydration and 12 h rehydration. (**A**) Survival rate of the glycerol treatment group. (**B**) The survival rate of the L-malic acid treatment group. (**C**) Survival rate of the KCl treatment group. (**D**) The survival rate of the sucrose treatment group. Lowercase alphabetical letters indicate a significant difference in the survival rate under different concentrations after dehydration treatment due to high osmotic pressure (*n* = 3, *p* < 0.05). Uppercase alphabetical letters indicate a significant difference in the survival rate after treatment with different osmolyte concentrations after rehydration (*n* = 3, *p* < 0.05). The error bars indicate a difference in the survival rate between the dehydration and the rehydration treatment same concentration (*n* = 3, * *p* < 0.05, ** *p* < 0.01). The color of the column differs based on the significance of survival rates.

The different effects of different concentrations of different osmolytes on the nematodes may lead to differences in the survival rate of nematodes. After staining nematodes with 0.5% methyl blue for 20 min, it was observed that a number of nematodes that had stopped moving were not stained under the microscope, indicating that these nematodes did not die but entered a dormant state after 6 h high osmotic pressure treatment. With the increase in osmolyte concentration, the number of stained nematodes increased, indicating that their mortality rate increased, and some of the nematode body fluids flowed out after treatment, indicating that the body cell walls of the nematodes were ruptured (Figure 3A–D and Figure 4). Moreover, different concentrations of osmolytes exhibited different effects on the survival rate of nematodes, as indicated by the different proportions of nematodes stained after the different treatments. When the treatment concentration was 10%, the number of stained nematodes in the L-malic acid treatment group was higher, the number of stained nematodes in the sucrose treatment group was lower, and there were no stained nematodes in the glycerol and KCl treatment groups. When the treatment concentration was 20%, stained nematodes could be observed after treatment with all four types of osmolytes for 6 h. Still, the number of stained nematodes in the L-malic acid treatment group was the highest, while the number of stained nematodes in the glycerol treatment group was the lowest (Figure 3E–G and Figure 4).

After 6 h of high osmotic pressure treatment with the four osmolyte types, it was found that the survival rate of nematodes decreased with the increase in osmolyte concentrations. Moreover, there were significant differences in the survival rates of nematodes treated with different osmolyte concentrations, with the highest survival observed in the glycerol treatment group (Figure 4A) and the lowest observed in the L-malic acid treatment group (Figure 4B). At 10–20% osmolyte treatment concentrations, the survival rates of nematodes in the glycerol, KCl, and sucrose treatment groups were all above 90% (Figure 4A,C,D), except for that in the L-malic acid treatment group, which resulted in a survival rate of less than 60% (Figure 4B). At 40% osmolyte treatment concentrations, the survival rate of nematodes began to decrease significantly, with the glycerol treatment group having the highest survival rate (>80%). In contrast, all nematodes in the L-malic acid treatment group died. When the osmolyte treatment concentration exceeded 50%, the survival rate of nematodes in all osmolyte treatment groups was less than 50%, except for glycerol. When the treatment concentration exceeded 70%, all the sucrose treatment group nematodes died. When the treatment concentration exceeded 90%, only a few nematodes in the glycerol treatment group survived, and most of them were DJ3. In addition, it was observed that the number of J2 and J3 after high osmotic pressure treatment decreased compared with their number when untreated. The concentration at which the number of J2 and J3 began to decrease was different for the different osmolyte types. It occurred at 80% L-malic acid and 90% glycerol, KCl, and sucrose concentrations, respectively. With a microscope, it was observed that this was due to the rupture of the body cell walls of J2 and J3 under high osmotic pressure until they disappeared completely.

After 6 h of high osmotic pressure treatment with four types of osmolytes and a subsequent rehydration treatment for 12 h, it was found that the survival rate of nematodes decreased after the rehydration treatment. Moreover, significant differences were observed in the survival rate of nematodes after rehydration. The survival rate of nematodes after rehydration in the glycerol treatment group was consistently higher compared to the other treatment groups (Figure 4A–D). The survival rate of nematodes after rehydration was the lowest in the L-malic acid treatment group. Under an osmolyte concentration of 20%, all nematodes in the L-malic acid treatment group died (Figure 4B). In osmolyte concentrations between 10 and 30%, except for the L-malic acid treatment group, the survival rate of nematodes after rehydration was decreased to a lower extent in the other treatment groups. When the osmolyte concentration ranged between 40 and 60%, the survival rate of nematodes after rehydration in the glycerol treatment group decreased significantly. However, it was still significantly higher than the other treatment groups (Figure 4A–D). When the osmolyte concentration was greater than 70%, all nematodes in the sucrose treatment group died after rehydration. However, the nematodes in the glycerol and KCl treatment group still survived after rehydration, and most of the surviving nematodes were DJ3.

Based on the PWN activity, phenotypic changes, and survival rate changes in the four osmolyte treatment groups, it could be concluded that the survival rate of PWN in the glycerol treatment group was generally higher. The PWN phenotypic changes were more obvious and easier to observe when treated with glycerol, so glycerol was more suitable for the further in-depth study of osmobiosis of PWN under high osmotic pressure. In addition, it was found that there were developmental stage-specific differences in PWNs under high osmotic stress, so in order to further study the mechanism of PWN resistance to high osmotic stress, glycerol was selected as the osmolyte inducing high-osmotic-pressure stress for further study.

### 3.3. Phenotypic Changes at Different PWN Developmental Stages under Glycerol-Induced High-Osmotic-Pressure Stress

**Figure 5:**
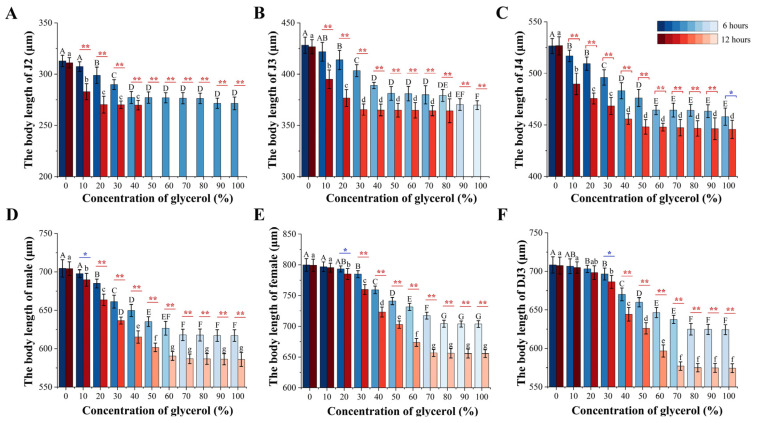
Changes in PWN body length with glycerol-induced high-osmotic-pressure stress for 6 h and 12 h. (**A**) J2. (**B**) J3. (**C**) J4. (**D**) Male. (**E**) Female. (**F**) DJ3. The uppercase alphabetical letters indicate significant differences in PWN body lengths after 6 h of high osmotic pressure treatment (*n* = 3, *p* < 0.05). The lowercase alphabetical letters indicate significant differences in PWN body lengths after 12 h of high osmotic pressure treatment (*n* = 3, *p* < 0.05). The error bars indicate differences in the PWN body length at different treatment times at the same glycerol concentration (*n* = 3, * *p* < 0.05, ** *p* < 0.01). The color of the column differs with the significance of body length differences.

Although the PWN body length decreased after 6 h or 12 h of glycerol treatment, there was no significant change in the PWN body width at each developmental stage. Therefore, the changes in PWN body length were used to explore the PWN phenotypic differences at each developmental stage under high osmotic pressure. The change in PWN body length varied greatly at different development stages at the same glycerol treatment concentration. There were no significant changes in body length for females at a glycerol concentration of 10% and for the DJ3 at a 10–20% concentration. In contrast, significant differences were observed in the body lengths in the other developmental stages at the other glycerol concentrations between 6 h and 12 h treatments (Figure 5). In the 6 h high-osmotic-pressure treatment group, the J2 nematodes began to disappear when the treatment concentration reached 90%, and J3 began to disappear when the treatment concentration reached 100%. Furthermore, in the 12 h high osmotic pressure treatment group, J2 began to disappear, when the treatment concentration exceeded 40%, and J3 began to disappear at treatment concentrations exceeded 80% (Figure 5A,B).

**Figure 6:**
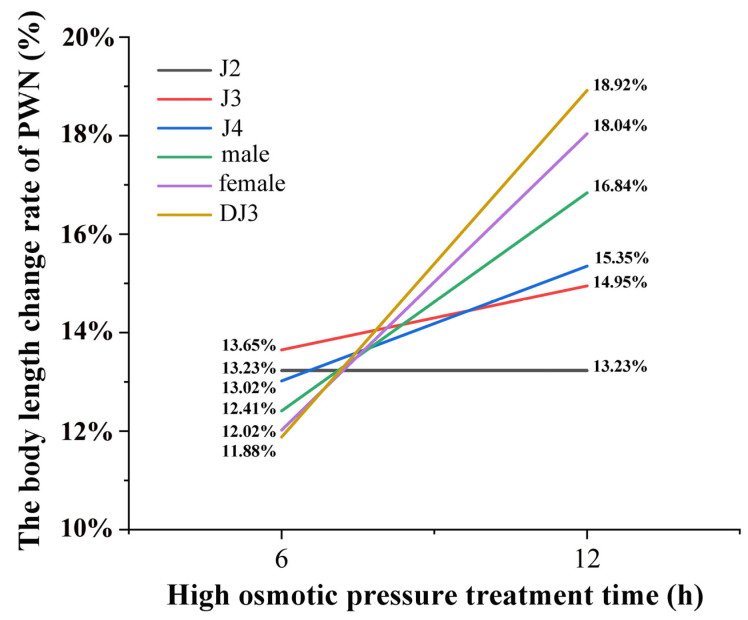
PWN body length change rate at each developmental stage after high osmotic pressure treatment. The change rate of body length was calculated based on the average body length (*n* = 30).

In addition, it was found that there were differences in the rate of change of body length at different developmental stages under the same treatment duration. In the 6 h high-osmotic-pressure treatment group, the DJ3 had the lowest change rate in body length and J3 the highest. Moreover, in the 12 h high-osmotic-pressure treatment group, J2 had the lowest change rate in body length and DJ3 the highest (Figure 6 and Table S2). Comparing the body length change rate between the 6 h and 12 h high osmotic pressure treatment groups, it was found that the body length change rate was increased in all developmental stages under the 12 h high osmotic pressure treatment, except for J2. Among them, the DJ3 exhibited the most increased body length change rate, at 18.92%. According to the changes in body length, the females were more suitable for the high osmotic stress environment than the males, and DJ3 was more suitable for the high osmotic stress environment than other juvenile stages. Moreover, J2 and J3 were the most difficult to survive in the high osmotic stress environment. As the body length change rate of DJ3 was greater than that of females, DJ3 exhibited a greater adaptation potential to high osmotic stress than females.

### 3.4. PWN Survival Rate Changes at Different Developmental Stages under Glycerol-Induced High-Osmotic-Pressure Stress

**Figure 7:**
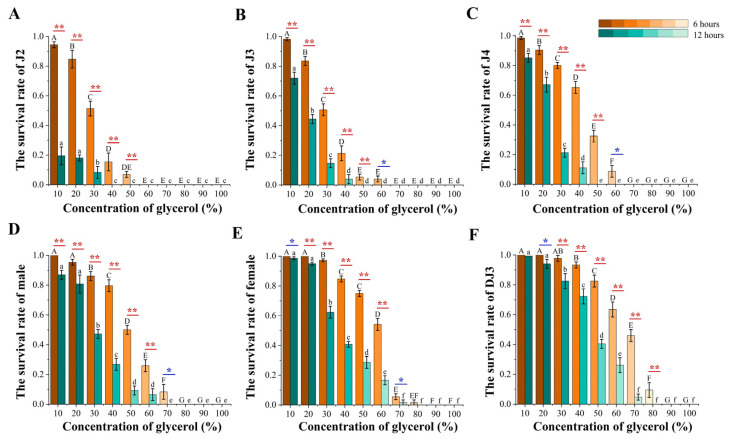
PWN survival rates after glycerol-induced high osmotic pressure treatment for 6 h and 12 h. (**A**) J2. (**B**) J3. (**C**) J4. (**D**) Male. (**E**) Female. (**F**) DJ3. The uppercase alphabetical letters indicate significant differences in the PWN survival rate after 6 h of high osmotic pressure treatment (*n* = 3, *p* < 0.05). The lowercase alphabetical letters indicate significant differences in PWN survival rates after 12 h of high osmotic pressure treatment (*n* = 3, *p* < 0.05). The error bars indicate differences in PWN survival rates at different treatment durations at the same concentration (*n* = 3, * *p* < 0.05, ** *p* < 0.01). The color of the column differs with the significance of the survival rate differences.

Except for the non-significant decrease in the survival rate of DJ3 at a 10% glycerol treatment concentration, the survival rate of nematodes at all developmental stages decreased after the high osmotic pressure treatment duration was increased from 6 h to 12 h. In the 6 h high osmotic pressure treatment group, all nematodes survived when the treatment concentration did not exceed 50%, all J2 died when the treatment concentration exceeded 50%, all J3 and J4 died when the treatment concentration exceeded 60%, all males died when the treatment concentration exceeded 70%, and all nematodes died when the treatment concentration exceeded 90%. In the 12 h high-osmotic-pressure treatment group, all nematodes survived when the treatment concentration did not exceed 30%, all J2 died when the treatment concentration exceeded 30%, all J3 and J4 died when the treatment concentration exceeded 40%, all males died when the treatment concentration exceeded 60%, and all nematodes died when the treatment concentration exceeded 70%. The survival rate of PWN was the lowest in J2 and the highest in DJ3 in both the 6 h and the 12 h high osmotic pressure treatment groups (Figure 7). Combined with the phenotypic changes in PWN at different developmental stages, a positive relationship was observed between the rate of body length change and the survival at each developmental stage in nematodes subjected to high-osmotic-pressure stress. Therefore, DJ3, exhibiting the highest survival rate, was selected to study the mechanism of PWN resistance to high osmotic pressure.

### 3.5. DGE Sequencing and Differently Expressed Genes Identification

**Figure 8:**
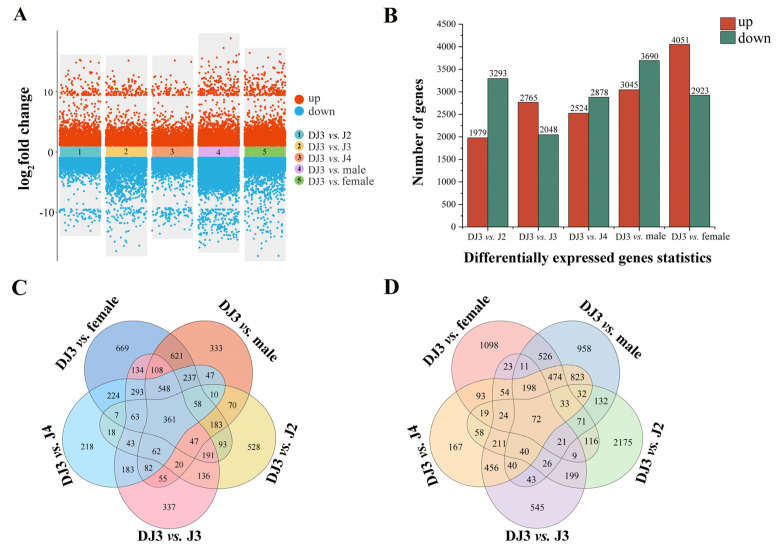
Gene expression analysis of PWN at different developmental stages. (**A**) Multi−group differential gene expression scatter plot at different developmental stages of PWN. (**B**) Statistics on the number of differentially expressed genes in DJ3. (**C**) Venn diagram of the up−regulated genes in DJ3. (**D**) Venn diagram of the down-regulated genes in DJ3.

In order to understand the molecular mechanism why DJ3 is more resistant to high osmotic stress than PWN at other developmental stages, transcriptome analysis was performed on PWN at different developmental stages, and the differentially expressed genes between DJ3 and PWN at other developmental stages were comparatively analyzed. Six PWN samples (J2, J3, J4, DJ3, female, male) were assessed by RNA-Seq sequencing technology, and an average of 23,374,559 original reads were obtained. After removing low-quality, adapter-sequence-containing reads or reads with high-N content corresponding to undetermined base N, the average number of clean reads remaining was 23,261,116 (Table S3). The average alignment rate between clean reads and the reference genome was 95.68%, and a total of 17,131 genes were identified. Fragments per kilobase of the exon model per million mapped fragments (FPKM) were used to normalize the gene expression to obtain non-repetitive gene expression, and the edgeR was used to screen the DEGs in DJ3 (Figure 8A). Compared with J2, 1979 genes were up-regulated, and 3293 genes were down-regulated in DJ3. When compared with J3, 2765 genes were up-regulated, and 2048 genes were down-regulated in DJ3. When compared with J4, 2524 genes were up-regulated, and 2878 genes were down-regulated in DJ3. When compared with males, 3045 genes were up-regulated, and 3690 genes were down-regulated in DJ3. When compared with females, 4051 genes were up-regulated, and 2923 genes were down-regulated in DJ3 (Figure 8B). In addition, 361 genes were uniquely up-regulated (Figure 8C), and 72 genes were uniquely down-regulated in DJ3 (Figure 8D).

### 3.6. Kyoto Encyclopedia of Genes and Genomes Enrichment Analysis Results of DEGs in DJ3

The genes that were differentially expressed only in DJ3 were analyzed for KEGG pathway enrichment, and the rich factor in each pathway was obtained. A higher rich factor corresponds to a greater degree of enrichment of the DEGs in the corresponding pathway [33]. The genes up-regulated only in DJ3 were enriched in a total of 188 pathways. Among them, there were 57 enriched pathways belonging to the metabolism category, including 17 pathways belonging to the carbohydrate metabolism subclass, which included 32 DEGs (Figure S1). The enriched pathways were arranged in an ascending order based on their *Q* value from low to high, and it was found that 5 of the top 20 most enriched pathways belonged to carbohydrate metabolism, having a rich factor value greater than 0.1 (Figure S2 and Table S4). They were all related to energy metabolism and the synthesis process of organic substances. These five pathways were pyruvate metabolism (Ko00620), propanoate metabolism (Ko00640), glycolysis/gluconeogenesis (Ko00010), glyoxylate and dicarboxylate metabolism (Ko00630), and fructose and mannose metabolism (Ko00051, Figure S3). Further analysis showed that the number of DEGs related to acetyl-CoA synthetase (ACS) was the highest among the DEGs enriched in these five pathways. These seven identified DEGs are involved in the acetyl-CoA biosynthesis pathway within the cell.

The genes uniquely down-regulated in DJ3 were enriched in 142 pathways. These included 21 pathways in the environmental information processing category, including 20 pathways in the signal transduction sub-class, with 3 enriched DEGs (Figure S4). The enriched pathways were arranged in an ascending order based on their *Q* value from small to large, which revealed that 12 of the top 20 pathways belonged to signal transduction, with rich factor values of higher than 0.01 (Figure S5 and Table S5). Among them, there were 6 pathways related to the smooth operation of cell physiological activities and adaptive response to adversity (Figure S6), including the JAK-STAT signaling pathway (ko04630), AMPK signaling pathway (ko04152), MAPK signaling pathway (ko04010), ErbB signaling pathway (ko04012), FoxO signaling pathway (ko04068), and HIF-1 signaling pathway (ko04066). Further analysis showed that the *AKT-1* gene was enriched in all six pathways, and it was the only enriched DEG identified.

### 3.7. Candidate Gene Screening and Expression Analysis

**Figure 9:**
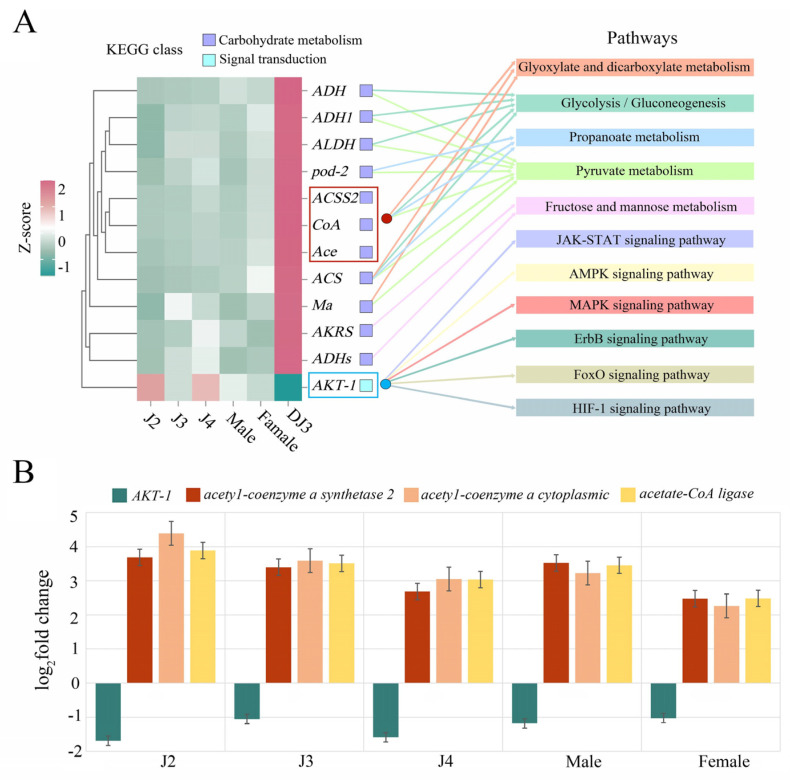
Candidate gene screening and expression patterns. (**A**) Candidate gene expression analysis. The left side illustrates the candidate gene heat map, and the right side illustrates the pathways enriched with the candidate genes (detailed in Table S6). (**B**) Expression validation of the selected genes by RT−qPCR (detailed in Table S7).

By analyzing the enrichment results of the DEGs in DJ3, the up-regulated genes enriched in five carbohydrate metabolism pathways related to the synthesis of energy compounds and the down-regulated genes enriched in six signal transduction pathways related to cellular stress resistance were selected for further expression analysis. Based on the RNA-seq results, the up-regulated *ACSS2*, *coA*, and *Ace* genes had similar expression patterns, and all three were ACS-pathway-related genes (Figure 9A, red box). These three genes were enriched in the glycolysis/gluconeogenesis, glyoxylate and dicarboxylate metabolism, pyruvate metabolism, and propanoate metabolism pathways, which are key pathways for the synthesis of metabolites involved in cellular energy homeostasis. In addition, the only gene enriched in the six signal transduction pathways was the *AKT-1* gene (Figure 9A, blue box). Therefore, these four genes (*ACSS2*, *coA*, *Ace*, and *AKT-1*) were selected for further analysis. RT-qPCR further verified the expression patterns of these four genes. The expression of *ACSS2*, *coA*, and *Ace* in DJ3 was significantly higher compared to other developmental stages, and the expression levels of *AKT-1* were significantly lower compared to other developmental stages, similar to the expression patterns obtained by the RNA-seq analyses (Figure 9B).

## 4. Discussion

Osmotic regulation is an important mechanism for plant resistance to adversity stress [34]. During osmoregulation, plants synthesize and accumulate large amounts of organic and inorganic compounds in their tissues. Their concentration in the cytosol increases, which results in an increase in osmotic pressure and the maintenance of turgor pressure to maintain normal cellular physiological processes [35]. In turn, the increased concentration of osmoregulatory compounds in tree sap and the elevated osmotic pressure can be detrimental to PWN, which invades the tree tissues. In adverse environments, some nematodes can reduce their water content, slow their metabolism, stop moving, and become coiled during osmobiosis. It was discovered that PWNs can stop their activities in adverse environments and only resume their activity when the environmental conditions are suitable for survival [18]. In a study on the environmental adaptability of *Caenorhabditis elegans* (Émile Maupas,1900), two strategies have been identified to resist adversity and enter osmobiosis. One is to enter a resting state, that is, cryptobiosis or diapause. The other may require high metabolic rates, osmotic regulation, and DNA repair while remaining active so that the cryptobiotic worms can withstand the damage caused by extreme environments [36]. In the staining experiment, the PWNs treated with the four osmolytes under high osmotic pressure did not die, although they were stationary, and they returned to an active state after the osmotic pressure was restored. This aligns with the osmobiosis phenomenon described in the literature [37], indicating that PWN may enter osmobiosis under high osmotic pressure. Therefore, under adverse environmental conditions, the elevated osmotic pressure of the tree’s tissues and sap may cause the PWN to enter osmobiosis. The nematode may return to normal physiological activity when the environment in which the tree is located is suitable and the osmotic pressure of the tree’s sap returns to normal.

In addition, the number of PWN and the developmental stage at which they entered osmobiosis under the high osmotic pressure treatment of four osmolytes differed. The nematodes in the L-malic acid treatment group entered osmobiosis at the earliest, across all stages of development. In the other three osmolyte treatments, compared to the sucrose treated group, all-developmental stages of PWN entered osmobiosis more slowly in the KCL and glycerol treatment groups. At 40% concentration, all developmental stages entered osmobiosis, but the proportion of females and DJ3 entering osmobiosis was smaller. Under a high osmotic pressure treatment, the volume of lipid droplets in nematodes entering osmobiosis and the number of large-volume lipid droplets increased. Studying the cryptobiosis phenomenon in *C. elegans* dauer, it was also found that the biomass density of *C. elegans* entering the diapause stage increased significantly due to the accumulation of lipid droplets [38]. Moreover, under high osmotic pressure, the larger the volume of lipid droplets in PWN, the more the number of large-volume lipid droplets, and the lower the survival rate of PWN. In the osmobiosis adaptation at the DJ3, a certain correlation was also observed between lipid droplets and nematode survival rates [18]. At lower treatment concentrations, the lipid droplets of PWNs treated with the four osmolytes had the largest volume, and the largest number of large-volume lipid droplets, and the disordered distribution of lipid droplets was the L-malic acid treatment group. In the glycerol treatment group, the volume of lipid droplets in PWN was the smallest, the lowest number of large-volume lipid droplets was observed, and the lipid droplets were closely arranged. The survival rate was calculated after 6 h of high osmotic pressure treatment. PWNs exhibited the highest survival rate in the glycerol treatment group, while the lowest survival rate was observed in the L-malic acid treatment group. Therefore, L-malic acid may exhibit toxicity on PWN. The changes in the volume of lipid droplets and the increase in the number of large-volume lipid droplets may be a special physiological adaptation of PWN in response to adverse environments.

In addition, PWNs tolerated up to 90% of the maximum concentration under high osmolarity treatment with a gradient concentration of pure glycerol (survival rate of 0.8%, and the surviving nematodes were at the DJ3). The PWNs were more active at higher treatment concentrations (40%) in the glycerol-treated group. Therefore, glycerol was confirmed to be a suitable osmolyte for studying PWN’s phenotypic changes and survival at each developmental stage under high-osmotic-pressure stress. Based on the survival rate of PWNs at different developmental stages under high osmotic pressure induced by glycerol, it was found that the DJ3 had the highest survival rate, followed by females. Regarding the phenotypic changes observed, the PWN body length change rate was the highest at the DJ3 and the lowest in J3, while no changes were observed in J2. This may indicate that the body cell membranes of DJ3 may have a higher flexibility, while J2 and J3 have a lower flexibility. In addition, observing phenotypic changes, it was also found that the body cell rupture in J2–J4 disappeared due to increased environmental osmotic pressure and longer osmotic pressure treatment duration. When the glycerol concentration was more than 90%, and the duration of high osmotic pressure treatment was 12 h, J2 and J3 could not be observed under the stereomicroscope. J4 were also reduced, but some J4 could still be observed under a stereomicroscope. J2–J4 correspond to the reproductive cycle of PWN with a suitable environment [39]. Compared with adults and DJ3, the fat content is lower, making it difficult to adapt to harsh environments [1]. In J2, the development rate of lipid droplets was slower than in adults. Usually, the presence of lipid droplets in adults reaches 58%, and lipid droplets appear in J2 [40]. When J2 are full of lipid droplets (as well as in the adults), it is considered that J2 are transformed into the DJ3. DJ3 are developed from J2, which contain a dense arrangement of lipid droplets in the body, which helps to resist adverse environments [40]. Therefore, the disappearance of J2-J4 may be due to the lower body fat content and the poor flexibility of the body cell membranes, resulting in the inability to adapt to high-osmotic-pressure stress. However, DJ3 have a higher content of fat in the body and a high flexibility of the cell membranes, which increases DJ3 ability to resist osmotic stress. DJ3 is characterized by a diapause state that can promote survival for a long time under adverse conditions. It is an important stage for PWN to survive under adverse environmental conditions [41]. Since J2, which can be converted to DJ3, are less resistant to osmotic stress, it is hypothesized that the high osmotic stress tolerance of the nematode can be utilized to control PWN.

To further explore the survival mechanism of the DJ3 under high osmotic stress and clarify the reasons for the differences observed among the PWN developmental stages under high osmotic stress, the gene expression of PWN at different developmental stages was analyzed. The results showed that the genes that were uniquely up-regulated in DJ3 were involved in carbohydrate metabolic processes such as glycolysis/gluconeogenesis, glyoxylate, dicarboxylate, pyruvate, propanoate, etc. Additionally, these pathways were enriched in ACS-pathway-related genes. ACS can convert acetate to acetyl coenzyme A (Acetyl CoA) [42]. Acetyl CoA is a key enzyme in the tricarboxylic acid cycle [43]. The tricarboxylic acid cycle is the core pathway for the metabolism of various energy compounds in the body [44]. Energy compounds are oxidized in the tricarboxylic acid cycle to produce carbon dioxide and water, releasing energy. At the same time, the intermediate products of the tricarboxylic acid cycle are also important raw materials for the biosynthesis of sugars, amino acids, fats, etc. [45]. The expression of ACS-related genes among the genes uniquely up-regulated in DJ3 was significantly higher compared to other developmental stages. Therefore, the acetyl CoA synthesis rate in DJ3 may be greater than the other developmental stages, which improves the body’s metabolic efficiency and promotes the accumulation of energy compounds such as sugars and fats.

Genes uniquely down-regulated in DJ3 were involved in the JAK-STAT signaling pathway, AMPK signaling pathway, MAPK signaling pathway, ErbB signaling pathway, FoxO signaling pathway, and other cell stress resistance-related pathways. The *AKT-1* gene was enriched in these pathways. AKT-1 is one of three closely related serine/threonine-protein kinases (AKT-1, AKT-2, and AKT-3), termed AKT kinases, which regulate many processes, including metabolism, proliferation, cell survival, growth, and angiogenesis [46]. Among the pathways enriched with *AKT-1*, the AMPK signaling pathway is an important signaling pathway in cell homeostasis maintenance. Adenosine 5′-monophosphate (AMP)-activated protein kinase (AMPK) plays a key role in many cells’ proliferation-related signaling pathways [47]. AMPK is a complex heterotrimer comprising an α catalytic subunit and two β and γ regulatory subunits. Its associated physiological processes are divided into five categories: protein metabolism, lipid metabolism, glucose metabolism, autophagy, and mitochondrial homeostasis [48]. Under adverse environmental conditions, AMPK regulates metabolism by regulating biosynthetic processes, inhibiting the synthesis of lipids, glycogen, and proteins, and reducing ATP consumption [49]. To store more ATP in order to maintain the body’s normal operation, AMPK can also increase glucose utilization through phosphorylation, increase the utilization of cell-stored lipids, and accelerate the conversion of macromolecules released by autophagy [50]. Phosphorylation of Thr172 is required for AMPK allostery during AMPK activation [51]. However, the AKT-1 protein phosphorylates specific ser487 in the ST loop of the α subunit, inhibiting the phosphorylation of Thr172, resulting in the inhibition of AMPK activation [52]. Therefore, compared with other developmental stages, the low expression of *AKT-1* may be helpful for DJ3 to trigger the AMPK signaling pathway quickly under adverse conditions, thus promoting a timely response to the damage caused by osmotic stress.

## 5. Conclusions

In summary, this study found that changes in environmental conditions can cause an increase in osmotic pressure in the host tree, while PWN can utilize the phenomenon of cryptobiosis to adapt to the high osmotic pressure environment. Under a high osmotic pressure environment, the different developmental stages of PWN differed significantly in terms of adaptation capacity. Among them, DJ3 had higher survival and body length change rates. By further analysis of gene expression, we found that the high survival rate of DJ3 may be related to the high expression of ACS-related genes, which promotes the accumulation of energy-related compounds, such as fat. At the same time, the low expression of *AKT-1* may contribute to the rapid activation of the AMPK signaling pathway, promoting the timely response to the damage from high osmotic pressure. In addition, it was found that glycerol was an ideal osmolyte suitable for studying the cryptic phenomenon of PWN under high osmotic pressure, and glycerol can be used to study further the molecular mechanisms of resistance to high-osmotic-pressure stress in the DJ3 of PWN. These findings offer a novel research foundation for further exploring the osmobiosis phenomenon of PWN. They also provide novel insights and methods for preventing and controlling PWN, which is of great significance for protecting forest resources and maintaining ecological security.

## Data Availability

The dataset was deposited in the Sequence Read Archive (BioProject ID: PRJNA1063576).

## Supplementary Materials

The following supporting information can be downloaded at: https://www.mdpi.com/article/10.3390/biology13020123/s1, Figure S1: KEGG enrichment result of genes only up-regulated in DJ3; Figure S2: KEGG enrichment result (top 20) for genes only up-regulated in DJ3; Figure S3: The network diagram of carbohydrate metabolism pathways; Figure S4: KEGG enrichment result of genes only down-regulated in DJ3; Figure S5: KEGG enrichment result (top 20) for genes only down-regulated in DJ3; Figure S6: The network diagram of signal transduction pathways; Table S1: The primers used in this study; Table S2: Body length changes of PWN at different developmental stages after high osmotic pressure treatment; Table S3: Statistical analysis of the RNA-seq data for each sample; Table S4: KEGG enrichment result (top 20) for genes only up-regulated in DJ3; Table S5: KEGG enrichment result (top 20) for genes only down-regulated in DJ3; Table S6: The candidate genes; Table S7: RT-qPCR results of selected genes.
