# Supplementary material for: Differences of Pine Wood Nematode (Bursaphelenchus xylophilus) Developmental Stages under High-Osmotic-Pressure Stress
Source: Biology (Basel). 2024 Feb 16;13(2):123. doi: 10.3390/biology13020123 (PMC10886877; doi:10.3390/biology13020123)
Supplement: Supplementary file 1 [file biology-13-00123-s001.zip › biology-2845284-supplementary.pdf]

Differences of Pine Wood Nematode(*Bursaphelenchus xylophilus*) Developmental Stages Under High-Osmotic-Pressure Stress

Shuting Wang [1,†], Qiaoli Chen [1,2,†,*], and Feng Wang [1,2,*3]

[1] Key Laboratory of Alien Forest Pests Monitoring and Control-Heilongjiang Province, School of Forestry, Northeast Forestry University, Harbin 150040, China; 1639788062@nefu.edu.cn (S.W.)

[2] Key Laboratory of Sustainable Forest Ecosystem Management-Ministry of Education, Northeast Forestry, University, Harbin 150040, China; fengwang@nefu.edu.cn (F.W.)

[3] State Key Laboratory of Tree Genetics and Breeding, Northeast Forestry University, Harbin 150040, China; fengwang@nefu.edu.cn (F.W.)

[*] Correspondence: qiaolichen@nefu.edu.cn (Q.C.); fengwang@nefu.edu.cn (F.W.)

[†] These authors contributed equally to this work.

Table S1. The primers used in this study.

Gene	Primer
acetyl-coenzyme a synthetase 2	F-`TGGACGACCTCATGAACGTG`<br>R-`CAAAGGCGTATGGGGAATGC`
acetyl-coenzyme a cytoplasmic	F-`TCATACCGTCGTCTTTGCCG`<br>R-`GAGTCCGGGCTTGGATCATT`
acetate--CoA ligase	F-`TTGGCAGGTCGTCGAAAAGT`<br>R-`TGTCAGGGAAACCCATCAGC`
AKT-1 protein	F-`CTTCAACGATGCAAGCACCC`<br>R-`AACATAGCCGATCCACCGTC`
18S ribosomal RNA	F-`CGAAGACGATCAGATACCGTCCTA`<br>R-`TTTCTCATAAGGTGCTGGCGGAGT`

Table S2. Body length changes of PWN at different developmental stages after high osmotic pressure treatment.

Treatment time (hour)	Developmental stage	Length of control group (μm)	Minimum length (μm)	Body length change rate
6	J2	312.93±5.59 (307.34～318.52)	271.53±6.19 (277.72～265.34)	13.23%
	J3	428.26±7.86 (436.12～420.40)	369.79±4.23 (374.09～365.49)	13.65%
	J4	526.51±7.27 (533.78～519.24)	457.99±8.30 (466.29～449.69)	13.02%
	Male	704.55±11.28 (715.83～693.27)	617.11±7.54 (625.31～610.23)	12.41%
	Female	799.89±10.21 (807.43～792.35)	703.74±5.43 (709.17～698.31)	12.02%
	DJ3	708.36±10.58 (718.94～697.78)	624.24±6.36 (630.6～617.88)	11.88%
12	J2	311.11±4.96 (316.07～306.15)	269.79±4.61 (274.40～265.18)	13.23%
	J3	426.81±6.87 (433.68～419.94)	364.23±11.64 (375.87～352.59)	14.95%
	J4	526.69±8.80 (535.49～517.89)	445.70±8.85 (454.55～436.85)	15.35%
	Male	704.15±8.87 (713.02～695.28)	585.92±9.06 (594.98～576.86)	16.84%
	Female	799.40±9.89 (809.29～789.51)	655.61±6.36 (661.97～649.25)	18.04%
	DJ3	707.50±11.01 (718.51～696.49)	574.37±5.79 (580.16～568.58)	18.92%

Table S3. Statistical analysis of the RNA-seq data for each sample.

Sample	Raw reads number	Clean reads number	Clean data rate (%)	Reference gene ratio (%)	Reference genome comparison ratio (%)
J2	22,217,822	22,133,728	99.62	71.27	95.87
J3	24,136,024	24,057,741	99.67	75.77	96.05
J4	24,136,024	24,037,515	99.59	76.96	95.75
DJ3	24,135,023	24,018,211	99.51	75.94	95.49
Male	21,485,349	21,359,122	99.41	76.86	95.34
Female	24,137,113	23,960,379	99.26	78.06	95.63

Table S4. KEGG enrichment result (top 20) for genes only up-regulated in DJ3.

KEGG A class	KEGG B class	Pathway	Pathway ID	Count
Metabolism	Global and overview maps	Metabolic pathways	ko01100	79
Metabolism	Xenobiotics biodegradation and metabolism	Metabolism of xenobiotics by cytochrome P450	ko00980	20
Metabolism	Xenobiotics biodegradation and metabolism	Drug metabolism - cytochrome P450	ko00982	17
Human Diseases	Cancer: overview	Chemical carcinogenesis - DNA adducts	ko05204	15
Metabolism	Carbohydrate metabolism	Pyruvate metabolism	ko00620	12
Metabolism	Xenobiotics biodegradation and metabolism	Drug metabolism - other enzymes	ko00983	18
Metabolism	Carbohydrate metabolism	Glycolysis / Gluconeogenesis	ko00010	12
Human Diseases	Cancer: overview	Chemical carcinogenesis - reactive oxygen species	ko05208	18
Metabolism	Carbohydrate metabolism	Glyoxylate and dicarboxylate metabolism	ko00630	7
Metabolism	Carbohydrate metabolism	Propanoate metabolism	ko00640	7
Metabolism	Metabolism of cofactors and vitamins	Ubiquinone and other terpenoid-quinone biosynthesis	ko00130	6
Human Diseases	Cancer: overview	Chemical carcinogenesis - receptor activation	ko05207	15
Metabolism	Metabolism of other amino acids	Glutathione metabolism	ko00480	10
Human Diseases	Cardiovascular disease	Fluid shear stress and atherosclerosis	ko05418	12
Metabolism	Global and overview maps	Carbon metabolism	ko01200	12
Human Diseases	Drug resistance: antineoplastic	Platinum drug resistance	ko01524	9
Organismal Systems	Aging	Longevity regulating pathway - worm	ko04212	10
Human Diseases	Cancer: specific types	Hepatocellular carcinoma	ko05225	11
Metabolism	Carbohydrate metabolism	Fructose and mannose metabolism	ko00051	6
Cellular Processes	Transport and catabolism	Lysosome	ko04142	20

Table S5. KEGG enrichment result (top 20) for genes only down-regulated in DJ3.

KEGG A class	KEGG B class	Pathway	Pathway ID	Count
Organismal Systems	Endocrine system	Renin-angiotensin system	ko04614	5
Organismal Systems	Immune system	Hematopoietic cell lineage	ko04640	3
Cellular Processes	Cell growth and death	Apoptosis	ko04210	2
Cellular Processes	Cell growth and death	Cellular senescence	ko04218	1
Cellular Processes	Cellular community eukaryotes	Adherens junction	ko04520	1
Cellular Processes	Cellular community eukaryotes	Focal adhesion	ko04510	2
Cellular Processes	Cellular community eukaryotes	Signaling pathways regulating pluripotency of stem cells	ko04550	2
Cellular Processes	Transport and catabolism	Lysosome	ko04142	5
Environmental Information Processing	Membrane transport	ABC transporters	ko02010	1
Environmental Information Processing	Signal transduction	AMPK signaling pathway	ko04152	1
Environmental Information Processing	Signal transduction	Apelin signaling pathway	ko04371	2
Environmental Information Processing	Signal transduction	Calcium signaling pathway	ko04020	1
Environmental Information Processing	Signal transduction	ErbB signaling pathway	ko04012	1
Environmental Information Processing	Signal transduction	FoxO signaling pathway	ko04068	1
Environmental Information Processing	Signal transduction	HIF-1 signaling pathway	ko04066	1
Environmental Information Processing	Signal transduction	Hippo signaling pathway	ko04390	1
Environmental Information Processing	Signal transduction	Hippo signaling pathway - fly	ko04391	1
Environmental Information Processing	Signal transduction	JAK-STAT signaling pathway	ko04630	1
Environmental Information Processing	Signal transduction	MAPK signaling pathway	ko04010	1
Environmental Information Processing	Signal transduction	Phospholipase D signaling pathway	ko04072	2

Table S6. The candidate genes.

Gene ID	Name	Homologous protein GenBank ID	Blast nr	*p*-value
BXY_0417300.1	*ADH*	XP_013031846.1	PREDICTED: alcohol dehydrogenase [NADP (+)] [*Anser cygnoides domesticus*]	4.67E-08
BXY_1767700.1	*ADH1*	NP_505991.1	Alcohol dehydrogenase 1 [*Caenorhabditis elegans*]	4.34E-96
BXY_0267600.1	*ALDH*	KHN86680.1	Aldehyde dehydrogenase, mitochondrial [*Toxocara canis*]	0
BXY_1014500.1	*Ma*	NP_503306.1	Malate synthase [*Caenorhabditis elegans*]	0
BXY_1230200.1	*ACSS2*	XP_001900923.1	acetyl-Coenzyme A synthetase 2 [*Brugia malayi*]	8.19E-73
BXY_0461400.1	*Ace*	ERG80270.1	acetyl-coenzyme a cytoplasmic [*Ascaris suum*]	4.11E-53
BXY_1230100.1	*ACS*	EJW73448.1	acetyl-CoA synthetase [*Wuchereria bancrofti*]	3.02E-23
BXY_1369600.1	*CoA*	KJH44216.1	acetate--CoA ligase [*Dictyocaulus viviparus*]	1.21E-75
BXY_1487400.1	*pod-2*	KKA67036.1	pod-2 [*Pristionchus pacificus*]	4.27E-14
BXY_0702500.1	*ADHs*	CEF69673.1	Alcohol dehydrogenase [NADP (+)] [*Strongyloides ratti*]	1.10E-39
BXY_0417800.1	*AKRS*	XP_013302589.1	oxidoreductase, aldo/keto reductase family protein [*Necator americanus*]	5.08E-31
BXY_0357600.1	*AKT-1*	AFY98834.1	AKT-1 protein [*Bursaphelenchus xylophilus*]	0

Table S7. RT-qPCR results of selected genes.

Gene ID	Name	log_2(DJ3/J2)	log_2(DJ3/J3)	log_2(DJ3/J4)	log_2(DJ3/Male)	log_2(DJ3/Female)
		J2	J3	J4	Male	Female
BXY_1230200.1	*acetyl-coenzyme a synthetase 2*	3.68736689	3.395723315	2.684630396	3.524224974	2.474998838
BXY_0461400.1	*acetyl-coenzyme a cytoplasmic*	4.392913146	3.592917275	3.047902283	3.22473448	2.260173296
BXY_1369600.1	*acetate--CoA ligase*	3.887966216	3.510710329	3.035982446	3.454341566	2.483059608
BXY_0357600.1	*AKT-1 protein*	-1.689187711	-1.054706796	-1.586709928	-1.183289552	-1.028565343

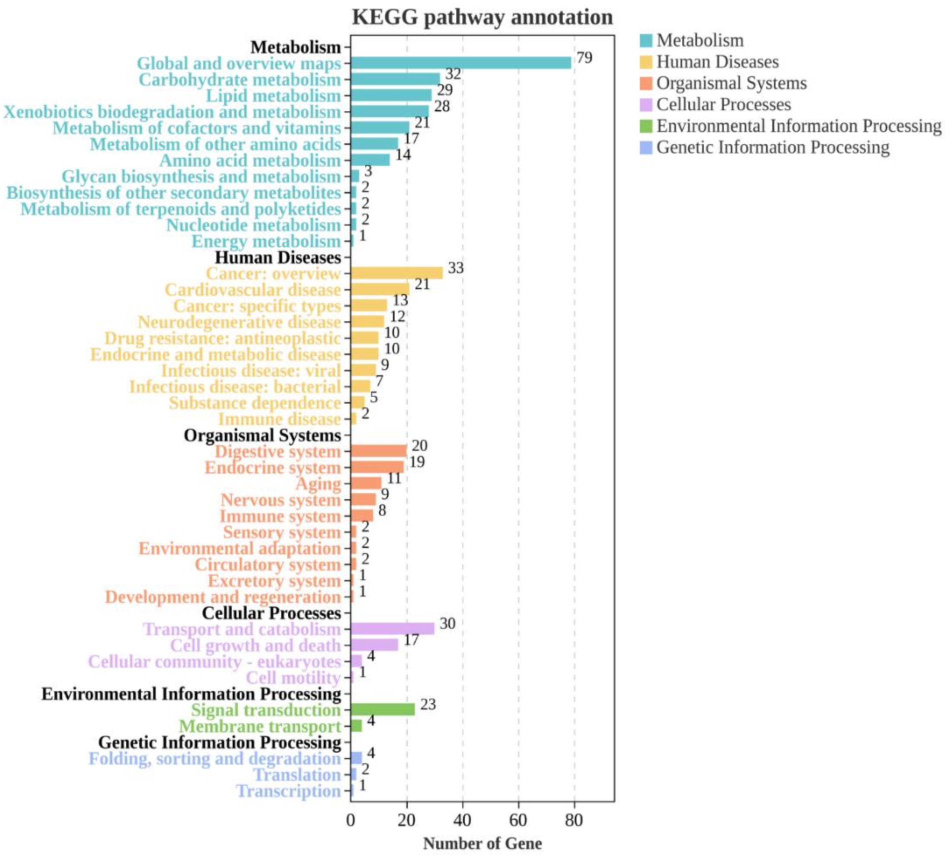

Figure S1. KEGG enrichment result of genes only up-regulated in DJ3.

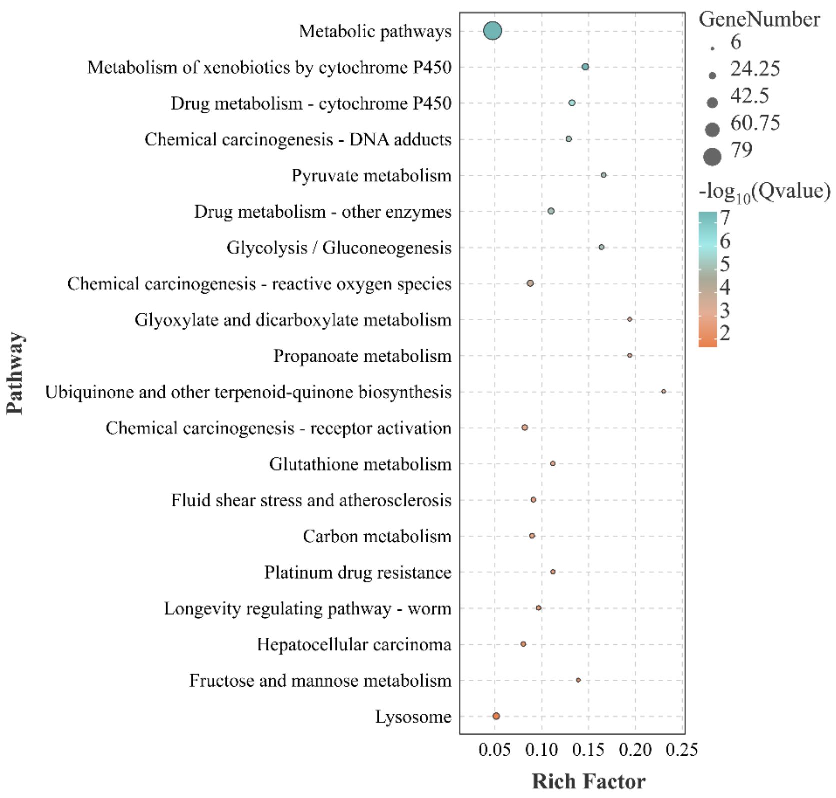

Figure S2. KEGG enrichment result (top 20) for genes only up-regulated in DJ3.

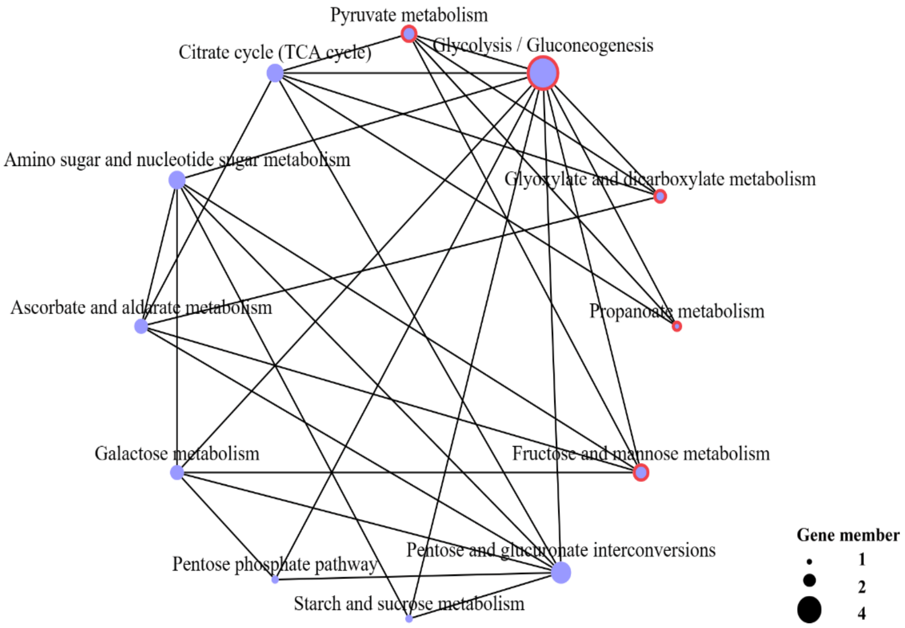

Figure S3. The network diagram of carbohydrate metabolism pathways. Red marked as selected pathways.

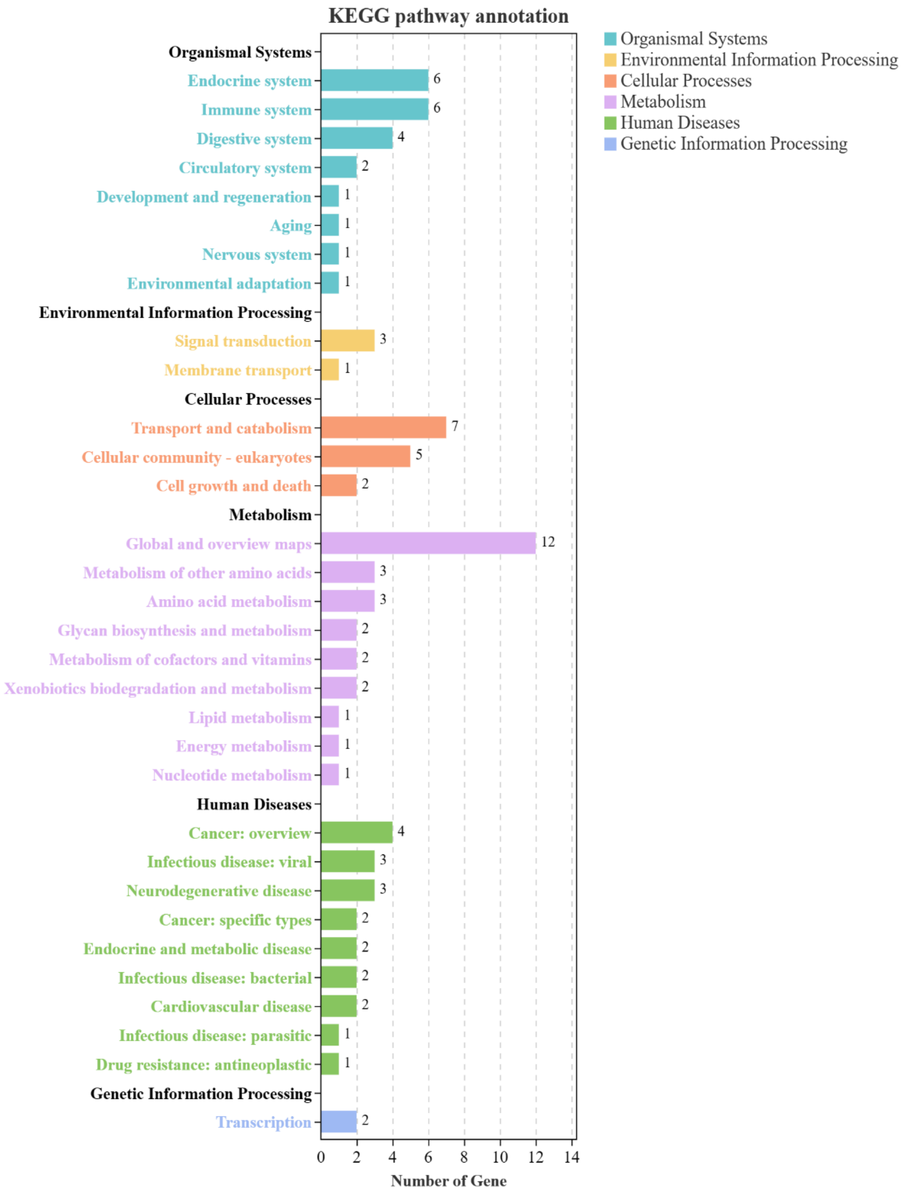

Figure S4. KEGG enrichment result of genes only down-regulated in DJ3.

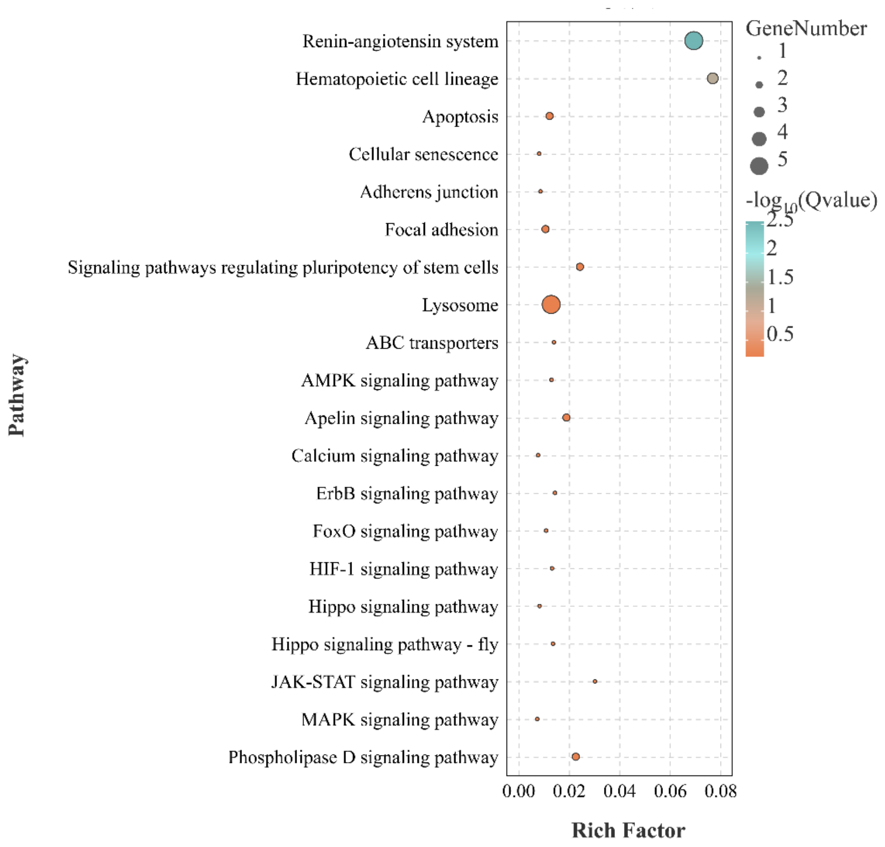

Figure S5. KEGG enrichment result (top 20) for genes only down-regulated in DJ3.

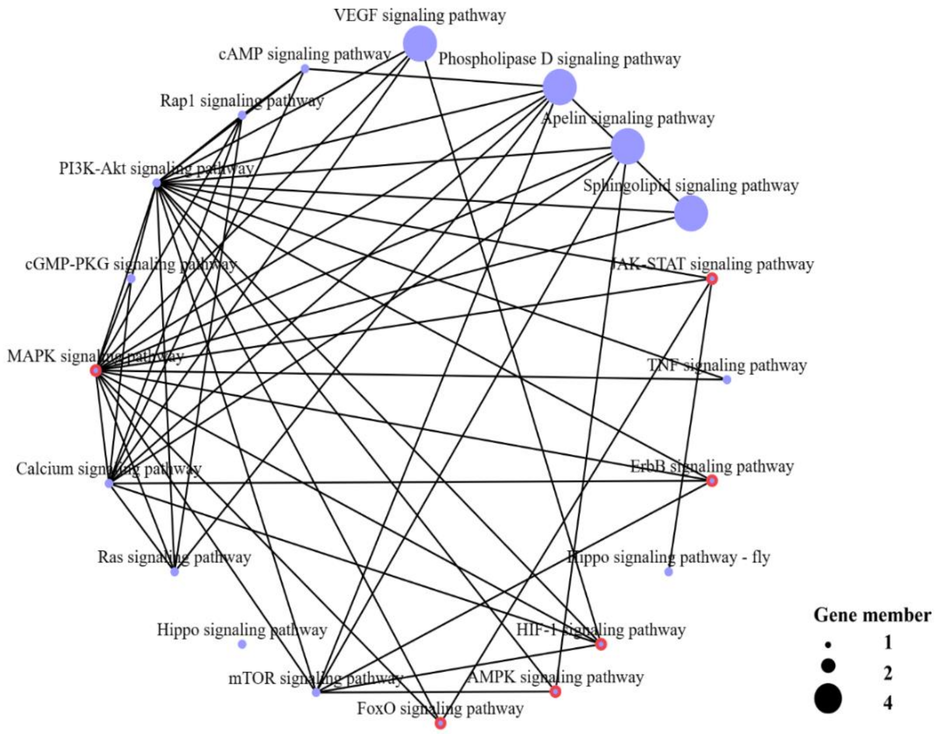

Figure S6. The network diagram of signal transduction pathways.
Red marked as selected pathways.
